# Associations of Adherence to the 2018 World Cancer Research Fund and the American Institute for Cancer Research Dietary Recommendations with Gut Microbiota and Inflammation Levels

**DOI:** 10.3390/nu15173705

**Published:** 2023-08-24

**Authors:** Dan Wang, Sijia Meng, Jiqiu Li, Jing Zhao, Yu Wang, Meizhi Du, Yuan Wang, Wenli Lu, Yun Zhu

**Affiliations:** Department of Epidemiology and Health Statistics, School of Public Health, Tianjin Medical University, Tianjin 300070, China; wd15122021812@163.com (D.W.); mengsijia@tmu.edu.cn (S.M.); 13821682687@163.com (J.L.); zhaojing201920@163.com (J.Z.); wangyu27wy@tmu.edu.cn (Y.W.); meizhi.du@tmu.edu.cn (M.D.); wangyuan@tmu.edu.cn (Y.W.); luwenli@tmu.edu.cn (W.L.)

**Keywords:** diet, WCRF/AICR, colorectal cancer, gut microbiota, inflammation, adenoma

## Abstract

Background: Whether the World Cancer Research Fund and the American Institute for Cancer Research (WCRF/AICR) dietary recommendations affect the gut microbiota and inflammatory status remains unclear. We examined the association of dietary adherence scores to the WCRF/AICR with gut microbiota and inflammation in a cross-sectional setting. Methods: The WCRF/AICR diet adherence scores were calculated for 151 participants (adenoma 97, non-adenoma 54) from 7-day dietary records. The gut microbiota was analyzed by 16S rRNA gene sequencing of fecal samples. The levels of inflammatory biomarkers in both blood (i.e., IL-6, IL-8, IgA, IgM, and IgG) and fecal samples (i.e., FCP) were evaluated in 97 colorectal adenoma patients who had blood samples available. Multivariable linear regression analyses were conducted to examine the association of individual and total dietary adherence scores with gut microbiota and inflammatory biomarker levels. Results: Participants with higher adherence had lower relative abundance of *Proteobacteria* (*β* = −0.041, 95%*CI*: −0.073, −0.009), *Enterobacteriaceae* (*β* = −0.035, 95%*CI*: −0.067, −0.003), and unidentified *Enterobacteriaceae* at the genus level (*β* = −0.029, 95%*CI*: −0.055, −0.003) compared to those with lower adherence. Plant-based food intake was positively correlated with increased abundance of *Phascolarctobacterium* (*β* = 0.013, 95%*CI*: 0.001, 0.026). Restricting fast food was linked to high abundance of *Bacteroidaceae* (*β* = 0.149, 95%*CI*: 0.040, 0.257) and *Bacteroides* (*β* = 0.149, 95%*CI*: 0.040, 0.257). Limiting sugary drinks was associated with reduced abundance of *Lachnospiraceae* (*β* = −0.155, 95%*CI*: −0.292, −0.018). Plant-based food intake (*β* = −0.251, 95%*CI*: −0.450, −0.052) and restriction of fast food (*β* = −0.226, 95%*CI*: −0.443, −0.008) were associated with reduced IGG levels in men. Alcohol restriction was linked to lower IL-6 (*β* = −7.095, 95%*CI*: −11.286, −2.903) and IL-8 (*β* = −7.965, 95%*CI*: −14.700, −1.230) levels in women, but with higher IL-6 (*β* = 0.918, 95%*CI*: 0.161, 1.675) levels in men. Conclusions: Our findings support the association of adherence to the WCRF/AICR diet with gut microbiota and inflammation. These results need to be validated in additional prospective or interventional studies.

## 1. Introduction

Colorectal cancer (CRC) is a global public health concern, ranking as the third most prevalent malignancy in men and the second in women, while also being the second leading cause of cancer-related mortality worldwide [1]. The adenoma–carcinoma sequence, with adenomas as the most frequent precancerous lesions, currently serves as the classical carcinogenic pathway explaining the majority of CRC cases [2,3]. Notably, modifiable environmental factors, such as dietary habits and lifestyles, contribute to the increasing incidence of CRC due to the ongoing trend towards westernized diets and sedentary behaviors [3].

Extensive research into the etiology of colorectal cancer has shed light on the crucial role of gut microbial composition and inflammation in the intricate relationship between diet and cancer [4]. The gut microbiota, influenced by dietary factors, acts as a key mediator in the interaction between the immune system and the host’s overall health and disease outcomes [5]. Consequently, therapeutic approaches targeting the gut microbiota and immune system through nutritional interventions, specifically diet modifications, have emerged as potential strategies for combating CRC [6].

In 2018, the World Cancer Research Fund and American Institute for Cancer Research released their updated 10 cancer prevention recommendations (2018 WCRF/AICR), providing evidence-based guidelines for maintaining a healthy diet, incorporating supplements, breastfeeding, weight management, and engaging in physical activity [7]. Studies have indicated that adhering to these recommendations not only enhances the quality of life for colorectal cancer survivors but also improves patient-reported outcomes following a cancer diagnosis [8,9,10,11]. The WCRF/AICR dietary recommendations, recognized as an anti-inflammatory diet, have demonstrated promising effects on human metabolism and immune function [12,13]. Previous investigations have shown that adherence to the 2007 WCRF/AICR dietary recommendations and energy-related dietary guidelines correlates with reduced oxidative stress and a more favorable inflammatory biomarker profile [14,15]. Furthermore, Laura et al. identified that higher consumption of animal-derived foods, processed foods, sugar, and alcohol coincided with a microbiome signature indicative of inflammation and was associated with elevated levels of intestinal inflammatory biomarkers, while plant-based foods were linked to a lower abundance of pathobionts [16].

Despite these findings, no studies have specifically examined the potential impact of diets adhering to the 2018 WCRF/AICR recommendations on gut microbiota composition and inflammatory pathways. Therefore, this study aimed to examine the association between adherence to these recommendations and gut microbiota composition, as well as its effects on inflammation in high-risk individuals for CRC, considering potential gender differences. Understanding this relationship could offer insights into CRC prevention and management strategies.

## 2. Materials and Methods

### 2.1. Study Design and Population

The present study used cross-sectional data obtained from the Colorectal Cancer Screening Project at Tianjin Nankai Hospital. All participants underwent colonoscopies between January 2019 and January 2021. The sample population consisted of 151 participants, of which 97 patients with colorectal adenoma were diagnosed by colonoscopy and 54 healthy volunteers without any colorectal cancer or precancerous/adenomatous lesions identified during the same period (non-adenomatous population). All participants signed informed consent.

A structured questionnaire was used to collect information from participants on (1) socio-demographic characteristics and anthropometric measures (e.g., age, gender); (2) selected lifestyle habits (e.g., smoking, alcohol drinking, yogurt consumption, and physical activity); (3) disease and health status (e.g., presence of comorbidities (including hypertension, hyperlipidemia, diabetes, coronary heart disease, gout, fatty liver, atherosclerosis, chronic gastroenteritis, appendicitis, duodenal ulcer), medication history, and self-reported height and weight); and (4) the utilization of dietary and health supplements. Body mass index (BMI) was calculated as weight divided by height^2^ (kg/m^2^). Participants’ adherence scores to the WCRF/AICR dietary recommendations were calculated from a detailed, consecutive 7-day dietary record, completed by each participant at the time of enrollment. Seven-day dietary records have been widely used to assess the nutrient intake of participants in epidemiological studies [17,18,19]. To minimize the possibility of information bias, participants were not provided with the specific WCRF/AICR dietary recommendations while filling out the dietary record. In addition, participants were required to provide blood and stool samples for subsequent measurement of inflammatory biomarker levels (adenoma participants) and gut microbiota, respectively. This study was approved by the Ethics Committee of Tianjin Nankai Hospital (No. NKYY_YXKT_IRB_2021_048_01).

### 2.2. World Cancer Research Fund/American Institute for Cancer Research Diet Adherence Score

We assessed adherence to the 2018 WCRF/AICR dietary recommendations using the scoring criteria proposed by Shams-White and Esposito et al. [20,21]. Specifically, we focused on 5 dietary recommendations: (R1) eating a diet rich in vegetables, fruits, and whole grains, (R2) limiting consumption of fast foods and other processed foods high in fat, sugar, or starch content, (R3) limiting consumption of red and processed meat, (R4) limiting consumption of sugary drinks, and (R5) limiting alcohol consumption. R1 was split into two sub-recommendations (as suggested by the standard scoring system [20,21]): 1a for vegetables and fruits and 1b for whole grains. For each dietary recommendation, participants were assigned 1, 0.5, and 0 points for complete adherence, partial adherence, and non-adherence, respectively. For 1a and 1b, participants were given 0.5 points for complete adherence, 0.25 for partial adherence, and 0 for non-adherence. These scores were then summed to calculate the total WCRF/AICR diet adherence score (ranging from 0 to 5), with higher scores indicating greater adherence to the dietary recommendations.

### 2.3. Collection of Fecal and Blood Samples

Fresh fecal samples were collected using a stool collector, rapidly frozen in liquid nitrogen, and transferred to the laboratory within 48 h. Venous blood samples from adenoma patients were collected using vacuum anticoagulated (EDTA) blood collection tubes, centrifuged within 4 h of the collection according to standard procedures, and plasma components were isolated and stored at −80 °C for subsequent testing.

### 2.4. DNA Extraction

Using the PowerMax (stool/soil) DNA isolation kit (MoBio Laboratories, Carlsbad, CA, USA), total bacterial genomic DNAs were extracted from fecal samples. They were then refrigerated at 20 °C for future analysis. The quantity and quality of isolated DNAs were measured using an agarose gel electrophoresis and a NanoDrop ND-1000 spectrophotometer (Thermo Fisher Scientific, Waltham, MA, USA).

### 2.5. 16S rDNA Amplicon Pyrosequencing

The gut microbial species and abundance of participants were determined by 16S rRNA Illumina NovaSeq high-throughput sequencing technology. The V3-V4 region of the bacterial 16 S rRNA gene was amplified by PCR using forward primer 341F CCTACGGGNGGCWGCAG and reverse primer 805R GACTACHVGGGTATCTAATCC. For multiplex sequencing, sample-specific paired-end 6 bp barcodes were added to the TrueSeq adaptors. The PCR components consisted of 10 μL DNA Template, 25 μL of Phusion High-Fidelity PCR Master Mix, 3 μL DMSO, 3 μL (10 μM) of Forward and Reverse primer, and 6 μL ddH2O. The thermal cycling comprised initial denaturation, followed by 25 cycles of denaturation, annealing, and extension, and final extension. Purification of PCR products was performed using Agencourt AMPure XP Beads (Beckman Coulter, Indianapolis, IN, USA), and quantification was performed by the Qubit dsDNA HS Assay. Subsequently, the PCR products were sequenced using the Illumina NovaSeq6000 platform at D I J I Info Technology Co., Ltd. (Nanjing, China).

### 2.6. Sequence Analysis

The Quantitative Insights Into Microbial Ecology (QIIME, v1.9.0) pipeline was used to process the sequencing data, as described previously [22]. In brief, raw sequencing reads with exact barcode matches were assigned to corresponding samples and identified as valid sequences. The screening criteria for low-quality sequences were as follows [23,24]: sequences <150 bp in length, sequences containing ambiguous bases, sequences with an average Phred score of <20, and sequences with mononucleotide repeats of >8 bp. Vsearch V2.4.4 was used to assemble pairs of end reads (--fastq_mergepairs --fastq_minovlen 5) and to perform operational taxonomic unit (OTU) picking, including dereplication, clustering, and detecting chimeras [25]. Using the default parameters to select a representative sequence from each OTU, a Vsearch of representative sequences against the Greengeen database was used to classify OTUs.

After generating the OTU table, the abundance of each OTU and its classification were recorded for each sample. In all samples, OTUs containing <0.001% of the total sequence were discarded. As an averaged, rounded, rarefied OTU table, we minimized differences in sequencing depth between samples by averaging over a subset of 100 uniformly resampled OTUs below 90% of the minimum sequencing depth.

### 2.7. Bioinformatics Analysis

The OTU table in QIIME (Quantitative Insights Into Microbial Ecology, v1.8.0, http://qiime.org, last accessed on 1 January 2018) was used to calculate OTU-level alpha diversity indexes, including the Shannon diversity index, Simpson index, Chao1 index, and ACE index. The results of clustering and annotation of OTUs, based on taxonomic information, allowed statistical analysis of community composition at different taxonomic levels (e.g., phylum, family, and genus levels). We used an online website (https://www.bioinformatics.com.cn, last accessed on 10 July 2023) to generate bubble plots of the relative abundance of gut microbiota according to dietary adherence subgroups.

### 2.8. Assessment of Inflammatory Biomarkers

We evaluated both systemic inflammation and local intestinal inflammation by measuring the levels of five inflammatory biomarkers (i.e., interleukin-6 (IL-6), interleukin-8 (IL-8), immunoglobulin A (IgA), immunoglobulin M (IgM), immunoglobulin G (IgG)) in the blood, as well as fecal calprotectin (FCP). The concentrations of IgA, IgM, IgG, IL-6, and IL-8 were quantified in the serum sample using the Merck MILLIPLEX^®®^ Protein Multifactor Liquid Chromatography Panel (Human Cytokine Autoantibody Panel). The assay was performed by Beijing Huatai Yikang Biotechnology Co. The quantification of FCP was performed using the Calprotectin ELISA Assay Kit (Eagle BioSciences, NH, USA) through an enzyme-linked immunosorbent assay (ELISA) method.

## 3. Statistical Analysis

The highest total score for adherence to dietary recommendations was 5 (complete adherence). A cut-off of 3 was used to divide participants into two groups: a low adherence group (0 to <3 points) and a high adherence group (3 to <5 points). Each separate score and the total score of the dietary recommendations were also considered for analysis as continuous variables.

The distributions of biomarker concentrations were skewed, and thus, we performed a natural log transformation of all biomarkers before analysis. The main characteristics of participants were described using means and standard deviations or frequencies and percentages. The *t*-test (continuous variables) and the *χ*^2^ test (categorical variables) were used to test the differences in the distribution of each variable between the two groups. Multivariable linear regression models were used to assess overall adherence scores (categorical and continuous variables) and adherence scores for each of the individual dietary recommendations (continuous variables) with gut microbiota and inflammation. Potential confounding variables included in the analysis of the association between adherence scores and inflammation were gender, age, BMI category (underweight, normal, overweight, and obese), number of comorbidities (0, 1, ≥2), long-term use of anti-inflammatory drugs (yes/no), smoking status (current, former, never). In the analysis of adherence scores and gut microbiota, additional adjusted for adenoma (yes/no), yogurt consumption (yes/no), and physical activity (</≥150 min/week). Furthermore, drinking status was also modeled as a categorical variable (never drinking, current drinking, former drinking) in the adherence scores of the first four dietary recommendations.

We explored whether associations differed between men and women by stratifying the analyses by sex. All analyses were performed with SAS 9.3 (SAS Institute, Cary, NC, USA). *p* < 0.05 (two-sided) was considered statistically significant.

## 4. Results

A total of 151 participants with a mean age of 61 years were included. The mean adherence score to the WCRF/AICR diet recommendations was 3.5 (maximum score of 5 and minimum score of 2). Participants with higher levels of adherence were older, more likely to be female, never smoked, never drank alcohol, and had ≥2 comorbidities compared to those with lower levels of adherence (Table 1). No statistical differences emerged for the other factors considered.

The degree of adherence to the WCRF/AICR dietary recommendations ranged from 2.6% to 94.0% (Table 2). None of the separate dietary recommendations were entirely adhered to by all participants. A total of 49.7% met the recommended daily intake of 400 g of fruit and vegetables. Only four persons (2.6%) met the recommendation to consume 30 g/day of dietary fiber. The ranking of adherence to dietary recommendations from highest to lowest was as follows: limit consumption of sugar-sweetened drinks (94.0%), limit consumption of ultra-processed foods (75.5%), limit alcoholic drink consumption (60.9%), eat adequate vegetables and fruits (49.0%), limit intakes of red and processed meat (45.0%), and eat diets rich in dietary fiber (2.6%).

### 4.1. WCRF/AICR Dietary Adherence and Gut Microbiota

**Figure 1:**
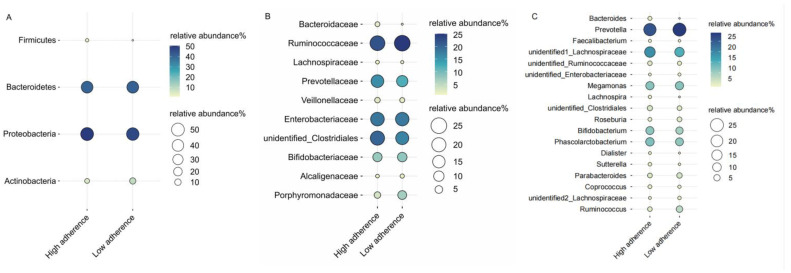
The distribution of gut bacteria’s relative abundance is depicted across phylum (**A**), family (**B**), and genus (**C**) levels, categorized by the WCRF/AICR dietary adherence scores. The size and color intensity of the circles represents the relative abundance (%) of the respective gut bacteria. Each gut bacteria’s name is presented on the left side, while the high/low adherence group label for each gut bacteria is displayed at the bottom.

We characterized the effect of adherence scores on the relative abundance of different bacterial taxa at the phylum, family, and genus levels. We restricted our analysis to gut microbiota with a relative abundance of 1% and higher. Examining the relative abundance of gut microbiota in relation to low and high adherence groups revealed that, at the phylum level, both groups were predominantly composed of *Proteobacteria* (48%) and *Bacteroidetes* (41%). At the family level, the high adherence group was marked by the dominance of *Ruminococcaceae* (22%) and an unidentified *Clostridiales* (20%), whereas the low adherence group was characterized by *Ruminococcaceae* (25%) and *Enterobacteriaceae* (18%). No discernible differences in microbiota composition between the two groups were observed at the genus level (Figure 1).

Using the low adherence group as a reference, the high adherence group exhibited a significant decrease in the relative abundance of *Proteobacteria* (*β* = −0.041, 95%*CI*: −0.073, −0.009) and *Enterobacteriaceae* (*β* = −0.035, 95%*CI*: −0.067, −0.003) (Table 3). Meanwhile, an unidentified *Enterobacteriaceae* had a lower relative abundance at the genus level (*β* = −0.029, 95%*CI*: −0.055, −0.003).

The results of the analysis of adherence to each dietary recommendation and gut microbiota are shown in Table 4. Higher adherence to vegetable, fruit, and whole grain recommendations was associated with a higher relative abundance of *Phascolarctobacterium* (*β* = 0.013, 95%*CI*: 0.001,0.026). Higher adherence to the fast food recommendations was positively correlated with the abundance of *Bacteroides* at both the family and genus levels (*β* = 0.149, 95%*CI*: 0.040, 0.257), while adherence to the sugary drinks recommendations was significantly associated with a decrease in the abundance of *Lachnospiraceae* (*β* = −0.155, 95%*CI*: −0.292, −0.018).

We further explored the potential relationship between the WCRF/AICR dietary adherence scores and the gut microbiota α-diversity (Shannon, Simpson, chao1, and Ace) indexes. However, our findings did not yield any significant results (Supplementary Tables S1 and S2).

### 4.2. WCRF/AICR Dietary Adherence and Inflammation

The overall WCRF/AICR diet adherence scores were not associated with the level of inflammation, with the low adherence group as the reference group, and this finding was consistent across gender stratifications. Furthermore, our analysis of adherence to the WCRF/AICR dietary recommendations as a continuous score yielded consistent findings compared to the results obtained from analyzing the exposures as categorical variables (Table 5).

In our analysis, we employed a gender-stratified approach and observed significant gender differences between adherence scores to each of the WCRF/AICR dietary recommendations and inflammatory biomarkers (Table 6). Specifically, the negative associations of IgG levels with adherence scores for recommendations on vegetables, fruits, and whole grains, as well as fast food consumption, were evident in men only (R1: *β* = −0.251, 95%*CI*: −0.450, −0.052; R2, *β* = −0.226; 95%*CI*: −0.443, −0.008). Moreover, limited alcohol consumption was positively correlated with IL-6 levels in men (*β* = 0.918, 95%*CI*: 0.161, 1.675) but conversely displayed a negative association in women (*β* = −7.095, 95%*CI*: −11.286, −2.093). Additionally, limited alcohol consumption was associated with lower IL-8 levels in women (*β* = −7.965, 95%*CI*: −14.700, −1.230).

## 5. Discussion

Understanding the role of the WCRF/AICR diet in modulating gut microbiota and inflammation can help identify new intervention targets, as well as optimize available methods to prevent or delay CRC. Our study stands as the only investigation exploring the relationship between the WCRF/AICR diet and gut microbiota. The key finding of our study was that adherence to the WCRF/AICR dietary recommendations had a significant effect on the relative abundance of gut microbiota; specifically, individuals with higher adherence to overall dietary recommendations showed a lower abundance of *Proteobacteria*, *Enterobacteriaceae*, and an unidentified *Enterobacteriaceae* at the genus level compared to those with lower adherence. Moreover, the intake of vegetables, fruits, and grains was positively associated with *Phascolarctobacterium* abundance. Limiting fast food consumption was associated with increased abundance of *Bacteroidaceae* and *Bacteroides*. In comparison, limiting sugary drinks was associated with decreased abundance of *Lachnospiraceae*. 

Recent studies have found that the *Proteobacteria* and *Enterobacteriaceae* were significantly increased in CRC samples compared to normal samples [26,27]. Numerous studies have demonstrated that the proliferation of potentially harmful *Proteobacteria*, especially *Enterobacteriaceae*, can lead to an increased inflammatory response in the host [28,29,30]. The *Enterobacteriaceae* family has been reported to interfere with intestinal metabolic processes, leading to alterations in the host’s bile acid (BA) metabolism [31], whereas reductions in BA levels seem to favor the growth of pathogenic and pro-inflammatory *Enterobacteriaceae* [32]. Thus, following the WCRF/AICR dietary recommendations can regulate the composition of the gut microbiota, probably by reducing the amounts of harmful or cancer-causing bacteria, such as *Proteobacteria* and *Enterobacteriaceae*. 

We observed that a higher intake of whole grains, fruits, and vegetables was associated with a higher abundance of *Phascolarctobacterium*. As a potentially beneficial bacterium, reduced numbers of *Phascolarctobacterium* were associated with the presence of colonic inflammation and disruption of gut homeostasis [33]. Higher contents of dietary fibers from fruits and vegetables have been reported to have beneficial effects on the gut microbiome in adults. Intervention studies conducted in both murine and human subjects have consistently reported a significant increase in the abundance of *Phascolarctobacterium* following soluble corn fiber supplementations [34,35]. In addition, higher dietary vitamin supplementation can also significantly increase the abundance of *Phascolarctobacterium* in the gut microbiota [36]. 

Our results indicated that adherence to the dietary recommendations for limiting the intakes of ‘fast food’ and other processed foods characterized by high fat, starch, or sugar content increased the abundance of both *Bacteroidaceae* and *Bacteroides* taxa in the gut microbiota. High-fat/carbohydrate diets have been reported to be associated with unfavorable changes in the gut microbiota, leaving it deficient in beneficial genera such as *Bacteroides* [37]. A systematic literature review on the effects of consuming ultra-processed, very-low-energy foods on the gut microbiota of obese patients found contradictory results that such diets may result in increased or decreased abundance from *Bacteroides* taxa [38]. Moreover, studies on Spanish populations observed that higher consumption of ultra-processed foods increased the abundance of *Bacteroidetaceae* in men but impacted differently in women [39]. Given the considerable individual variation in the response of the gut microbiota to dietary habits, the effects of different food groups, compositions, and contents on the human gut are complex. We also observed that limiting sugary drinks was associated with a reduced abundance of *Lachnospiraceae*, which is consistent with previous reports [16,40]. The progression of CRC may be affected by a higher abundance of the *Lachnospiraceae* family in the gut microbiome [41]. As previously reported, some *Lachnospiraceae* strains produce metabolites that are toxic or trigger ecological dysregulation in the host and may exert harmful effects on host health [42]. Their effects on host physiology remain variable and inconclusive across various investigations. 

The improved biomarker profile primarily resulted from following the guidelines for consuming vegetables, fruits, and whole grains, and limiting intake of processed foods and alcohol. In contrast, limiting red and processed meat intake and sugary drinks consumption did not appear to affect inflammatory status. Thus, different dietary habits may have important effects in determining the concentrations of biomarkers such as IL-6, IL-8, and IgG. Adherence to limit alcohol consumption and IL-6 levels showed inconsistent relationships across genders, with possible explanations due to the different types of alcohol consumed by men and women. As described in previous studies, distinct characteristics of drinking patterns (binge or regular) or different types of alcoholic drinks (wine, beer, hard liquor, or mixed) may lead to significant changes in biomarkers status and metabolic differences that predict adverse health outcomes [43]. Based on prior literature, the stimulation of oxidative stress and inflammatory consequences of excessive alcohol consumption may be influenced by gender-specific factors [44,45]. These results emphasize the sex-related differences in alcohol-induced inflammation or immune responses. The pro-inflammatory factor IL-6 plays a major role in the chronic inflammatory condition of the body, and its increased levels are thought to stimulate CRC progression [46], while previous studies have reported no significant association between adherence to the 2007 WCRF/AICR recommendations for limiting alcohol consumption and IL-6 [15]. This contradictory result may be attributed to the utilization of distinct operationalization criteria for alcohol consumption (ethanol intake vs. frequency of alcohol intake). IL-8 has been validated to have the potential to screen for CRC and its precancerous lesions [47]. We found that adherence to limiting alcohol consumption had a notable inhibitory impact on IL-8. These results are in line with a prior controlled human study that demonstrated a significant increase in IL-8 levels after alcohol consumption [48]. 

Our results showed that higher whole grains, vegetables, and fruit intakes were associated with lower IGG levels. Increased IgG in serum is usually accompanied by various inflammations in the body [49]. The presence of multiple bioactive compounds in fruits and vegetables was reported to have a protective effect in reducing the risk of developing non-communicable diseases due to chronic inflammation. Among them, dietary polyphenols have been identified as significant contributors [50], which affect immune function by affecting the synthesis of pro-inflammatory cytokines and gene expression [51]. In addition, we found that limiting the intake of high-energy processed foods was associated with a significant decrease in serum IgG levels. Consumption of ultra-processed foods may have an adverse effect on the inflammatory state [39,52]. An intervention study showed that controlling dietary energy intake led to a marked decrease in IgG, thereby enhancing anti-inflammatory responses and promoting overall immune system health [53]. Additionally, animal experiments have shown that a high-sugar and high-fat diet can significantly increase serum IGG levels and induce immune system dysfunction, resulting in chronic intestinal inflammation [49].

As we are aware, this is the first study examining the association of adherence to WCRF/AICR dietary recommendations with gut microbiota and inflammation, taking into account differences between gender in a Chinese population. A notable strength of this study is the use of a continuous 7-day dietary record approach that enables quantitative assessment of the precise intake of individual foods and nutrients. To elaborate, meticulous instructions on completing the food record were provided by the dietitians, including information about the detailed composition of each food item, as well as accurate assessments of portion sizes and units of food. Supplementary data were also requested from participants when required, thus enhancing the credibility and validity of the reported consumption of food and drink.

The current study is limited by the cross-sectional design, which hampers our analysis of the dynamic diet-microbiome relationship over time and the causal inferences regarding the effects of the WCRF/AICR diet on gut microbiota and inflammation. Addressing these constraints necessitates a long-term follow-up in longitudinal studies. The potential for reporting bias should be considered when interpreting self-reported dietary intake data, as participants tended to report food consumption close to social expectations [54]. For instance, there is a tendency to overestimate the intake of fruits, vegetables, and other health-conscious choices, while underestimating the consumption of energy-dense foods and alcohol. Hence, it is possible that adherence to dietary recommendations could be slightly overestimated. Moreover, a comprehensive investigation involving a broader range of inflammatory biomarkers (e.g., TNF-alpha and IL-17) has the potential to provide a deeper and more nuanced comprehension of the intricate relationship between the WCRF/AICR diet and inflammation. In addition, the lack of references in the literature on WCRF/AICR adherence scores with gut microbiota and inflammation may also make the selection of an appropriate cut-off point difficult, given that different grouping criteria may cause variations in the study results.

## 6. Conclusions

The present study suggested that adherence to the WCRF/AICR dietary recommendations may contribute to a favorable gut microbial environment and improve the inflammatory status of adenoma patients. Intervention studies are urgently needed in the future to verify whether adherence to these dietary recommendations can improve and fine-tune the quality of life and outcomes.

## Figures and Tables

**Table 1:** Characteristics of participants within categories of adherence to the WCRF/AICR diet score (*n* = 151). [n (%)/(Mean values ± standard deviations)].

	WCRF/AICR Diet Score in Categories		*p*
	Low Adherence (0 to <3 Points)	High Adherence (3 to <5 Points)	
N	41	110	
Age (years)	58.49 ± 7.53	62.39 ± 6.73	0.003
Sex (%)			0.001
Men	31(75.6)	50(45.4)	
Women	10(24.4)	60(54.6)	
BMI (kg/m^2^)	24.34 ± 3.06	24.00 ± 3.02	0.537
BMI (%)			0.858
Underweight (<18.5 kg/m^2^)	1(2.4)	5(4.6)	
Normal weight (18.5–24.9 kg/m^2^)	24(58.5)	63(57.3)	
Overweight (25–29.9 kg/m^2^)	14(34.2)	39(35.5)	
Obese (≥30 kg/m^2^)	2(4.9)	3(2.7)	
Smoking status (%)			0.002
Never	23(56.1)	85(77.3)	
Current	14(34.2)	11(10.0)	
Former	4(9.8)	14(12.7)	
Drinking status (%)			<0.0001
Never	13(31.7)	79(71.8)	
Current	20(48.8)	20(18.2)	
Former	8(19.5)	11(10.0)	
Moderate-to-vigorous physical activity (min/week) (%)			0.113
<150	32(78.0)	71(64.6)	
1150	9(22.0)	39(35.4)	
Comorbidities (%) *			0.002
0	15(36.6)	19(17.3)	
1	4(9.7)	39(35.4)	
92	22(53.7)	52(47.3)	
Yogurt consumption (%)			0.948
Yes	21(51.2)	57(51.8)	
No	20(48.8)	53(48.2)	
Long-term use of anti-inflammatory drugs (%)			0.267
Yes	24(58.5)	75(68.2)	
No	17(41.5)	35(31.8)	
Adenoma (%)			0.372
Yes	24(58.5)	73(66.4)	
No	17(41.5)	37(33.6)	

BMI: body mass index; * comorbidities include hypertension, hyperlipidemia, diabetes, coronary heart disease, gout, fatty liver, atherosclerosis, chronic gastroenteritis, appendicitis, and duodenal ulcer.

**Table 2:** Dietary WCRF/AICR cancer prevention recommendations and adherence in participants (*n* = 151) *.

2018 WCRF/AICR Recommendations ^a^	Operationalization/Comments	Score	Adherence	
			*n*	%
1. Eat a diet rich in whole grains, vegetables, and fruit	Fruits and vegetables (g/day)			
	≥400	0.5	75	49.7
	200 to <400	0.25	42	27.8
	<200	0	34	22.5
	Total fiber (g/day)			
	≥30	0.5	4	2.6
	15 to <30	0.25	28	18.5
	<15	0	119	78.8
2. Limit consumption of ‘fast foods’ and other processed foods high in fat, starches, or sugars	Percentage of total energy from adapted ultra-processed foods			
	Tertile 1	1	114	75.5
	Tertile 2	0.5	30	19.9
	Tertile 3	0	7	4.6
3. Limit consumption of red and processed meat	Total red meat (g/week) and processed meat (g/week)			
	Red meat <500 and processed meat <21	1	68	45.0
	Red meat <500 and processed meat 21 to <100	0.5	30	19.9
	Red meat ≥500 or processed meat ≥100	0	53	35.1
4. Limit consumption of sugar-sweetened drinks	Total sugar-sweetened drinks (g/day)			
	0	1	142	94.0
	0 to ≤250	0.5	9	6.0
	>250	0	0	0.0
5. Limit alcohol consumption	Alcoholic drinks (n/week)			
	0	1	92	60.9
	0 to ≤7	0.5	57	37.8
	>7	0	2	1.3

* According to the National Cancer Institute operationalization of the 2018 WCRF/AICR recommendations. ^a^ The optional recommendation on breastfeeding was not included. WCRF: World Cancer Research Fund. AICR: American Institute for Cancer Research.

**Table 3:** Differences in the relative abundance of gut microbiota among participants grouped according to the WCRF/AICR dietary adherence scores at the phylum, family, and genus levels (*n* = 151). (*β*-Coefficients and 95% confidence intervals).

Levels	Taxa	WCRF/AICR Diet Score		Continuous WCRF/AICR Diet Score
		Low Adherence (0 to <3 Points, *n* = 41)	High Adherence (3 to <5 Points, *n* = 110)	
Phylum	Firmicutes	reference	0.012(−0.054, 0.078)	0.011(−0.034, 0.055)
	Bacteroidetes	reference	0.005(−0.064, 0.073)	−0.005(−0.051, 0.041)
	Proteobacteria	reference	−0.041(−0.073, −0.009)	−0.009(−0.031, 0.013)
	Actinobacteria	reference	0.019(−0.007, 0.045)	0.001(−0.017, 0.019)
Family	Bacteroidaceae	reference	−0.013(−0.086, 0.061)	0.004(−0.046, 0.053)
	Ruminococcaceae	reference	0.007(−0.041, 0.054)	0.009(−0.023, 0.041)
	Lachnospiraceae	reference	0.005(−0.036, 0.046)	0.006(−0.022, 0.033)
	Prevotellaceae	reference	0.031(−0.066, 0.129)	0.006(−0.060, 0.071)
	Veillonellaceae	reference	0.007(−0.024, 0.038)	−0.001(−0.021, 0.020)
	Enterobacteriaceae	reference	−0.035(−0.067, −0.003)	−0.006(−0.028, 0.016)
	unidentified_Clostridiales	reference	−0.001(−0.011, 0.009)	−0.001(−0.007, 0.006)
	Bifidobacteriaceae	reference	0.016(−0.007, 0.039)	−0.001(−0.016, 0.015)
	Alcaligenaceae	reference	−0.002(−0.008, 0.004)	−0.001(−0.004, 0.004)
	Porphyromonadaceae	reference	0.006(−0.002, 0.015)	0.001(−0.005, 0.006)
Genus	Bacteroides	reference	−0.013(−0.086, 0.061)	0.004(−0.046, 0.053)
	Prevotella	reference	0.031(−0.066, 0.129)	0.006(−0.060, 0.071)
	Faecalibacterium	reference	0.003(−0.026, 0.031)	0.009(−0.010, 0.029)
	unidentified_Lachnospiraceae	reference	−0.002(−0.026, 0.022)	0.005(−0.011, 0.021)
	unidentified_Ruminococcaceae	reference	0.004(−0.020, 0.029)	0.003(−0.013, 0.020)
	unidentified_Enterobacteriaceae	reference	−0.029(−0.055, −0.003)	−0.006(−0.024, 0.012)
	Megamonas	reference	−0.012(−0.040, 0.015)	−0.010(−0.028, 0.009)
	Lachnospira	reference	0.010(−0.004, 0.023)	0.003(−0.007, 0.012)
	unidentified_Clostridiales	reference	−0.001(−0.011, 0.009)	−0.001(−0.007, 0.006)
	Roseburia	reference	−0.009(−0.020, 0.002)	−0.005(−0.012, 0.003)
	Bifidobacterium	reference	0.016(−0.007, 0.039)	−0.001(−0.016, 0.015)
	Phascolarctobacterium	reference	0.003(−0.026, 0.031)	0.009(−0.010, 0.029)
	Dialister	reference	0.001(−0.006, 0.007)	−0.001(−0.006, 0.003)
	Sutterella	reference	0.009(−0.003, 0.020)	0.002(−0.006, 0.010)
	Parabacteroides	reference	0.008(−0.001, 0.017)	0.004(−0.002, 0.010)
	Coprococcus	reference	0.005(−0.002, 0.013)	0.001(−0.004, 0.007)
	unidentified_Lachnospiraceae	reference	−0.002(−0.007, 0.002)	−0.001(−0.003, 0.002)
	Ruminococcus	reference	0.001(−0.006, 0.007)	−0.001(−0.006, 0.003)

WCRF/AICR: World Cancer Research Fund/American Institute for Cancer Research. Multivariable linear regression model adjusted for age, gender, adenoma (yes/no), BMI category, number of comorbidities (0, 1, ≥2), long-term use of anti-inflammatory drugs (yes/no), yogurt consumption (yes/no), smoking (never, ever, current), and physical activity (</≥150 min/week).

**Table 4:** Relationship between the WCRF/AICR dietary adherence scores and the relative abundance of gut microbiota at the phylum, family, and genus levels (*n* = 151). (*β*-Coefficients and 95% confidence intervals).

Levels	Taxa	WCRF/AICR Diet Score				
		R1-Vegetables, Fruits, and Whole Grains Intake	R2-Limit Fast Foods	R3-Limit Red and Processed Meat	R4-Limit Sugary Drinks	R5-Limit Alcohol
Phylum	Firmicutes	0.012(−0.084, 0.109)	0.033(−0.066, 0.133)	0.002(−0.059, 0.062)	−0.168(−0.391, 0.055)	0.053(−0.068, 0.173)
	Bacteroidetes	0.019(−0.081, 0.119)	−0.003(−0.107, 0.100)	0.002(−0.061, 0.065)	0.133(−0.099, 0.365)	−0.108(−0.232, 0.015)
	Proteobacteria	−0.042(−0.089, 0.006)	−0.031(−0.080, 0.019)	0.009(−0.021, 0.039)	0.011(−0.101, 0.122)	0.009(−0.051, 0.069)
	Actinobacteria	0.005(−0.034, 0.043)	−0.006(−0.045, 0.034)	−0.010(−0.034, 0.014)	0.004(−0.086, 0.094)	0.046(−0.002, 0.093)
Family	Bacteroidaceae	−0.070(−0.176, 0.037)	0.149(0.040, 0.257)	−0.027(−0.095, 0.040)	0.019(−0.232, 0.269)	0.021(−0.114, 0.155)
	Ruminococcaceae	0.033(−0.036, 0.103)	0.008(−0.064, 0.080)	0.000(−0.044, 0.044)	−0.057(−0.219, 0.105)	0.022(−0.065, 0.109)
	Lachnospiraceae	−0.016(−0.076, 0.044)	0.021(−0.041, 0.083)	0.005(−0.033, 0.042)	−0.155(−0.292, −0.018)	0.065(−0.010, 0.139)
	Prevotellaceae	0.088(−0.054, 0.229)	−0.123(−0.269, 0.023)	0.043(−0.047, 0.132)	0.086(−0.246, 0.419)	−0.110(−0.288, 0.068)
	Veillonellaceae	−0.005(−0.050, 0.040)	0.015(−0.032, 0.062)	−0.001(−0.030, 0.027)	0.052(−0.053, 0.157)	−0.026(−0.083, 0.030)
	Enterobacteriaceae	−0.037(−0.085, 0.010)	−0.032(−0.081, 0.017)	0.011(−0.019, 0.041)	0.028(−0.083, 0.140)	0.011(−0.049, 0.071)
	unidentified_Clostridiales	0.007(−0.007, 0.022)	−0.006(−0.021, 0.009)	−0.001(−0.010, 0.008)	−0.011(−0.045, 0.023)	0.004(−0.015, 0.022)
	Bifidobacteriaceae	0.005(−0.029, 0.038)	−0.008(−0.042, 0.027)	−0.009(−0.030, 0.012)	0.003(−0.075, 0.081)	0.034(−0.007, 0.076)
	Alcaligenaceae	−0.004(−0.012, 0.005)	0.004(−0.005, 0.013)	0.000(−0.005, 0.006)	−0.007(−0.027, 0.013)	0.000(−0.011, 0.011)
	Porphyromonadaceae	−0.001(−0.013, 0.012)	−0.002(−0.016, 0.011)	−0.002(−0.010, 0.006)	0.010(−0.020, 0.039)	0.010(−0.006, 0.026)
Genus	Bacteroides	−0.070(−0.176, 0.037)	0.149(0.040, 0.257)	−0.027(−0.095, 0.040)	0.019(−0.232, 0.269)	0.021(−0.114, 0.155)
	Prevotella	0.088(−0.054, 0.229)	−0.123(−0.269, 0.023)	0.043(−0.047, 0.132)	0.086(−0.246, 0.419)	−0.110(−0.288, 0.068)
	Faecalibacterium	0.031(−0.010, 0.073)	0.019(−0.024, 0.062)	0.003(−0.023, 0.029)	−0.052(−0.149, 0.045)	−0.004(−0.056, 0.048)
	unidentified_Lachnospiraceae	−0.014(−0.048, 0.021)	0.021(−0.015, 0.056)	0.006(−0.015, 0.028)	−0.079(−0.159, 0.001)	0.025(−0.018, 0.068)
	unidentified_Ruminococcaceae	−0.002(−0.037, 0.034)	−0.009(−0.046, 0.028)	0.005(−0.017, 0.028)	−0.011(−0.094, 0.073)	0.023(−0.022, 0.067)
	unidentified_Enterobacteriaceae	−0.036(−0.074, 0.003)	−0.022(−0.062, 0.018)	0.008(−0.016, 0.032)	0.017(−0.074, 0.108)	0.009(−0.040, 0.058)
	Megamonas	−0.002(−0.042, 0.038)	−0.015(−0.057, 0.026)	−0.003(−0.029, 0.022)	0.033(−0.060, 0.127)	−0.044(−0.094, 0.005)
	Lachnospira	0.004(−0.017, 0.024)	0.004(−0.017, 0.025)	0.002(−0.011, 0.015)	−0.024(−0.071, 0.023)	0.008(−0.017, 0.033)
	unidentified_Clostridiales	0.007(−0.007, 0.022)	−0.006(−0.021, 0.009)	−0.001(−0.010, 0.008)	−0.011(−0.045, 0.023)	0.004(−0.015, 0.022)
	Roseburia	−0.015(−0.031, 0.001)	−0.005(−0.022, 0.011)	−0.004(−0.014, 0.007)	−0.029(−0.066, 0.009)	0.019(−0.001, 0.039)
	Bifidobacterium	0.005(−0.029, 0.038)	−0.008(−0.042, 0.027)	−0.009(−0.030, 0.012)	0.003(−0.075, 0.081)	0.034(−0.007, 0.076)
	Phascolarctobacterium	0.013(0.001, 0.026)	0.012(−0.001, 0.026)	0.001(−0.007, 0.009)	−0.001(−0.031, 0.030)	−0.011(−0.027, 0.005)
	Dialister	0.002(−0.015, 0.019)	0.004(−0.014, 0.021)	0.000(−0.011, 0.011)	0.010(−0.029, 0.050)	0.004(−0.017, 0.025)
	Sutterella	−0.004(−0.012, 0.005)	0.005(−0.004, 0.014)	0.000(−0.005, 0.006)	−0.008(−0.029, 0.012)	0.000(−0.011, 0.011)
	Parabacteroides	−0.001(−0.013, 0.012)	−0.002(−0.016, 0.011)	−0.002(−0.010, 0.006)	0.010(−0.020, 0.039)	0.010(−0.006, 0.026)
	Coprococcus	0.009(−0.002, 0.020)	0.003(−0.009, 0.014)	−0.003(−0.010, 0.004)	−0.010(−0.036, 0.015)	0.008(−0.006, 0.021)
	unidentified_Lachnospiraceae	−0.002(−0.008, 0.004)	0.001(−0.005, 0.006)	0.001(−0.003, 0.004)	−0.009(−0.023, 0.005)	0.003(−0.005, 0.010)
	Ruminococcus	0.003(−0.006, 0.013)	0.001(−0.008, 0.011)	−0.006(−0.012, 0.000)	0.004(−0.018, 0.026)	0.004(−0.008, 0.016)

WCRF/AICR: World Cancer Research Fund/American Institute for Cancer Research. Multivariable linear regression model adjusted for age, gender, adenoma (yes/no), BMI category, number of comorbidities (0, 1, ≥2), long-term use of anti-inflammatory drugs (yes/no), yogurt consumption (yes/no), smoking (never, ever, current), and physical activity (</≥150 min/week). Multivariable models for R1 to 4 were additionally adjusted for drinking (never, former, current), except for R5.

**Table 5:** Association between adherence to the World Cancer Research Fund/American Institute for Cancer Research (WCRF/AICR) diet score and biomarkers of inflammation (*n* = 97). (*β*-Coefficients and 95% confidence intervals).

		WCRF/AICR Diet Score		Continuous WCRF/AICR Diet Score
Biomarkers		Low Adherence (0 to <3 Points, *n* = 24)	High Adherence (3 to <5 Points, *n* = 73)	
IL-6 (pg/mL)	Total (*n* = 97)	reference	0.143(−0.333, 0.619)	0.140(−0.174, 0.454)
	Men (*n* = 58)	reference	0.167(−0.270, 0.603)	0.071(−0.254, 0.396)
	Women (*n* = 39)	reference	0.259(−1.137, 1.654)	0.193(−0.564, 0.950)
IL-8 (pg/mL)	Total (*n* = 97)	reference	−0.062(−0.867, 0.743)	−0.097(−0.629, 0.435)
	Men (*n* = 58)	reference	−0.326(−1.220, 0.568)	−0.162(−0.827, 0.503)
	Women (*n* = 39)	reference	0.458(−1.602, 2.518)	−0.110(−1.234, 1.013)
IgA (g/L)	Total (*n* = 97)	reference	−0.111(−0.299, 0.078)	−0.024(−0.150, 0.102)
	Men (*n* = 58)	reference	−0.102(−0.344, 0.139)	−0.022(−0.202, 0.159)
	Women (*n* = 39)	reference	−0.188(−0.597, 0.220)	−0.035(−0.261, 0.190)
IgG (g/L)	Total (*n* = 97)	reference	−0.188(−0.597, 0.220)	−0.021(−0.081, 0.040)
	Men (*n* = 58)	reference	−0.052(−0.159, 0.054)	−0.047(−0.126, 0.032)
	Women (*n* = 39)	reference	−0.052(−0.223, 0.120)	−0.010(−0.104, 0.084)
IgM (g/L)	Total (*n* = 97)	reference	−0.039(−0.288, 0.211)	−0.038(−0.203, 0.127)
	Men (*n* = 58)	reference	−0.090(−0.384, 0.204)	−0.124(−0.340, 0.091)
	Women (*n* = 39)	reference	0.271(−0.318, 0.861)	0.069(−0.255, 0.393)
FCP (ug/g)	Total (*n* = 97)	reference	−0.519(−1.288, 0.251)	−0.239(−0.751, 0.273)
	Men (*n* = 58)	reference	−0.796(−1.735, 0.144)	−0.638(−1.331, 0.055)
	Women (*n* = 39)	reference	0.408(−1.219, 2.034)	0.526(−0.339, 1.390)

WCRF/AICR: World Cancer Research Fund/American Institute for Cancer Research. Multivariable linear regression model adjusted for age, gender, BMI category, number of comorbidities (0, 1, ≥2), long-term use of anti-inflammatory drugs (yes/no), and smoking (never, ever, current).

**Table 6:** Association between adherence to the World Cancer Research Fund/American Institute for Cancer Research (WCRF/AICR) diet score and biomarkers of inflammation (*n* = 97). (*β*-Coefficients and 95% confidence intervals).

		WCRF/AICR Diet Score				
Biomarkers		R1-Vegetables, Fruits, and Whole Grains Intake	R2-Limit Fast Foods	R3-Limit Red and Processed Meat	R4-Limit Sugary Drinks	R5-Limit Alcohol
IL-6 (pg/mL)	Total (*n* = 97)	−0.204(−0.895, 0.486)	−0.188(−0.949, 0.574)	0.318(−0.101, 0.738)	−0.007(−1.744, 1.731)	0.437(−0.491, 1.364)
	Men (*n* = 58)	−0.358(−1.165, 0.449)	−0.371(−1.235, 0.493)	0.080(−0.344, 0.505)	−0.400(−2.254, 1.455)	0.918(0.161, 1.675)
	Women (*n* = 39)	−0.883(−2.024, 0.258)	−0.239(−1.511, 1.032)	0.427(−0.388, 1.242)	−0.816(−3.951, 2.319)	−7.095(−11.286, −2.903)
IL-8 (pg/mL)	Total (*n* = 97)	0.086(−1.101, 1.273)	−0.695(−1.995, 0.605)	−0.092(−0.822, 0.637)	0.464(−2.515, 3.444)	0.278(−1.311, 1.867)
	Men (*n* = 58)	0.125(−1.652, 1.902)	−0.588(−2.481, 1.306)	−0.433(−1.353, 0.487)	0.603(−3.449, 4.655)	0.659(−0.976, 2.294)
	Women (*n* = 39)	−0.283(−2.197, 1.630)	−0.977(−2.990, 1.035)	−0.092(−1.428, 1.245)	−1.059(−6.106, 3.988)	−7.965(−14.700, −1.230)
IgA (g/L)	Total (*n* = 97)	−0.079(−0.354, 0.195)	−0.090(−0.392, 0.213)	−0.001(−0.170, 0.168)	0.049(−0.641, 0.739)	0.034(−0.338, 0.406)
	Men (*n* = 58)	−0.128(−0.602, 0.347)	−0.191(−0.697, 0.315)	−0.013(−0.261, 0.236)	0.356(−0.725, 1.437)	0.078(−0.366, 0.523)
	Women (*n* = 39)	−0.081(−0.503, 0.341)	0.045(−0.407, 0.497)	−0.017(−0.312, 0.278)	−0.314(−1.425, 0.797)	−0.019(−1.506, 1.467)
IgG (g/L)	Total (*n* = 97)	−0.094(−0.224, 0.036)	−0.068(−0.212, 0.076)	0.005(−0.076, 0.086)	−0.072(−0.402, 0.257)	0.070(−0.106, 0.246)
	Men (*n* = 58)	−0.251(−0.450, −0.052)	−0.226(−0.443, −0.008)	0.017(−0.095, 0.128)	−0.111(−0.597, 0.374)	0.078(−0.118, 0.274)
	Women (*n* = 39)	−0.067(−0.241, 0.107)	0.061(−0.126, 0.247)	−0.003(−0.126, 0.120)	−0.135(−0.597, 0.328)	−0.047(−0.666, 0.572)
IgM (g/L)	Total (*n* = 97)	−0.064(−0.428, 0.301)	0.013(−0.389, 0.415)	−0.054(−0.278, 0.170)	0.051(−0.865, 0.967)	−0.130(−0.619, 0.359)
	Men (*n* = 58)	−0.177(−0.759, 0.404)	−0.063(−0.687, 0.562)	−0.075(−0.380, 0.229)	−0.409(−1.736, 0.917)	−0.275(−0.809, 0.259)
	Women (*n* = 39)	0.080(−0.524, 0.684)	0.025(−0.621, 0.672)	0.059(−0.362, 0.480)	0.801(−0.765, 2.367)	0.754(−1.370, 2.879)
FCP (ug/g)	Total (*n* = 97)	−0.752(−1.874, 0.369)	0.140(−1.109, 1.390)	−0.237(−0.932, 0.458)	0.851(−1.989, 3.691)	0.083(−1.436, 1.602)
	Men (*n* = 58)	−1.255(−3.148, 0.639)	−0.545(−2.606, 1.516)	−0.703(−1.690, 0.284)	0.560(−3.846, 4.966)	−0.174(−1.948, 1.599)
	Women (*n* = 39)	−0.218(−1.880, 1.445)	0.933(−0.808, 2.674)	0.518(−0.625, 1.661)	1.363(−3.002, 5.727)	0.585(−5.264, 6.434)

WCRF/AICR: World Cancer Research Fund/American Institute for Cancer Research. Multivariable linear regression model adjusted for age, gender, BMI category, number of comorbidities (0, 1, ≥2), long-term use of anti-inflammatory drugs (yes/no), and smoking (never, ever, current). Multivariable models for R1 to 4 were additionally adjusted for drinking (never, former, current), except for R5.

## Data Availability

Data are available upon reasonable request.

## Supplementary Materials

The following supporting information can be downloaded at: https://www.mdpi.com/article/10.3390/nu15173705/s1. Table S1: Association between adherence to the World Cancer Research Fund/American Institute for Cancer Research (WCRF/AICR) diet score and gut microbiota diversity (*n* = 151). (β-Coefficients and 95 % confidence intervals). Table S2: Association between adherence to the World Cancer Research Fund/American Institute for Cancer Research (WCRF/AICR) diet score and gut microbiota α-diversity(*n* = 151). (β-Coefficients and 95 % confidence intervals).
