# Supplementary material for: Associations of Adherence to the 2018 World Cancer Research Fund and the American Institute for Cancer Research Dietary Recommendations with Gut Microbiota and Inflammation Levels
Source: Nutrients. 2023 Aug 24;15(17):3705. doi: 10.3390/nu15173705 (PMC10490500; doi:10.3390/nu15173705)
Supplement: Supplementary file 1 [file nutrients-15-03705-s001.zip › nutrients-2540703-supplementary.pdf]

Table S1: Association between adherence to the World Cancer Research Fund/American Institute for Cancer Research (WCRF/AICR) diet score and gut microbiota diversity (n=151). (β-Coefficients and 95 % confidence intervals)

		WCRF/AICR diet score		Continuous WCRF/AICR diet score
		Low adherence (0 to <3 points, n=41)	High adherence (3 to <5points, n=110)	
shannon	Total(n=151)	reference	-0.056(-0.322,0.209)	-0.035(-0.217,0.146)
	Male(n=81)	reference	-0.002(-0.368,0.364)	0.012(-0.257,0.281)
	Female(n=70)	reference	-0.240(-0.735,0.254)	-0.110(-0.371,0.151)
simpson	Total(n=151)	reference	0.0010(-0.0002,0.0003)	0.0000(-0.0002,0.0002)
	Male(n=81)	reference	0.0001(-0.0003,0.0005)	0.0000(-0.0002,0.0003)
	Female(n=70)	reference	-0.0002(-0.0006,0.0003)	-0.0001(-0.0003,0.0002)
chao1	Total(n=151)	reference	-900.902(-1850.486,48.683)	-87.436(-728.349,553.477)
	Male(n=81)	reference	-881.463(-2128.547,365.621)	81.238(-788.406,950.882)
	Female(n=70)	reference	-1141.303(-3077.991,795.385)	-401.546(-1441.261,638.169)
ace	Total(n=151)	reference	-934.399(-1910.406,41.608)	-85.002(-722.382,574.378)
	Male(n=81)	reference	-959.146(-2247.815,329.523)	77.997(-821.688,977.681)
	Female(n=70)	reference	-1094.170(-3067.110,878.770)	-390.785(-1451.702,670.133)

WCRF/AICR: the World Cancer Research Fund/American Institute for Cancer Research. Multivariable linear regression model adjusted for age, adenoma(yes/no), bmi category, number of comorbidities (0, 1, ≥2), long-term use of anti-inflammatory drugs(yes/no), yogurt consumption(yes/no), smoking (never, ever, current), and physical activity (</ ≥ 150 min/week).

Table S2: Association between adherence to the World Cancer Research Fund/American Institute for Cancer Research (WCRF/AICR) diet score and gut microbiota α-diversity(n=151). (β-Coefficients and 95 % confidence intervals)

		WCRF/AICR diet score				
		R1- vegetables, fruits, and whole grains intake	R2-limit fast foods	R3-limit red and processed meat	R4- limit sugary drinks	R5-limit alcohol
shannon	Total(n=151)	-0.058(-0.453,0.337)	-0.172(-0.579,0.236)	0.059(-0.189,0.307)	-0.325(-1.245,0.595)	-0.058(-0.553,0.436)
	Male(n=81)	0.034(-0.615,0.682)	-0.491(-1.201,0.219)	0.140(-0.246,0.527)	-0.360(-1.714,0.994)	0.132(-0.533,0.798)
	Female(n=70)	-0.086(-0.641,0.469)	0.081(-0.440,0.602)	-0.092(-0.449,0.265)	-0.128(-1.661,1.404)	-0.372(-1.277,0.533)
simpson	Total(n=151)	-0.0001(-0.0005,0.0003)	-0.0001(-0.0005,0.0003)	0.0001(-0.0001,0.0004)	-0.0003(-0.0012,0.0006)	0.0001(-0.0004,0.0005)
	Male(n=81)	-0.0001(-0.0007,0.0006)	-0.0006(-0.0013,0.0002)	0.0003(-0.0001,0.0006)	-0.0006(-0.0020,0.0008)	0.0002(-0.0005,0.0009)
	Female(n=70)	-0.0001(-0.0006,0.0004)	0.0002(-0.0003,0.0007)	-0.0001(-0.0004,0.0002)	0.0002(-0.0012,0.0016)	-0.0002(-0.0011,0.0006)
chao1	Total(n=151)	-123.178(-1552.851,1306.494)	-924.086(-2394.288,546.116)	215.879(-683.399,1115.157)	444.979(-2890.566,3780.523)	-719.53(-2506.066,1067.006)
	Male(n=81)	305.856(-1935.068,2546.779)	-1630.504(-4088.093,827.084)	151.468(-1189.304,1492.240)	1275.704(-3407.036,5958.444)	-406.514(-2708.619,1895.590)
	Female(n=70)	-384.888(-2567.163,1797.387)	-338.071(-2385.1587,1709.016)	131.231(-1275.498,1537.961)	-808.653(-6829.791,5212.483)	-1709.832(-5258.266,1838.601)
ace	Total(n=151)	-135.119(-1604.985,1334.747)	-876.848(-2389.687,635.991)	218.043(-706.558,1142.643)	502.922(-2926.264,3932.107)	-839.823(-2675.365,995.720)
	Male(n=81)	317.368(-2001.963,2636.699)	-1675.940(-4219.988,868.108)	150.862(-1236.866,1538.590)	1311.247(-3535.500,6157.994)	-518.422(-2899.918,1863.075)
	Female(n=70)	-424.583(-2644.183,1795.016)	-208.709(-2292.717,1875.552)	124.910(-1306.216,1556.036)	-641.742(-6766.613,5485.129)	-1888.854(-5493.301,1715.592)

WCRF/AICR: the World Cancer Research Fund/American Institute for Cancer Research. Multivariable linear regression model adjusted for age, adenoma(yes/no), bmi category, number of comorbidities (0, 1, ≥2), long-term use of anti-inflammatory drugs(yes/no), yogurt consumption(yes/no), smoking (never, ever, current), and physical activity (</ ≥ 150 min/week). Multivariable models for scores 1 to 4 were additionally adjusted for drinking (never, former, current), except for score 5.
